# Early social environment affects attention to social cues in juvenile common ravens, *Corvus corax*

**DOI:** 10.1098/rsos.220132

**Published:** 2022-06-29

**Authors:** Mario Gallego-Abenza, Palmyre H. Boucherie, Thomas Bugnyar

**Affiliations:** ^1^ Department of Behavioral and Cognitive Biology, University of Vienna, Vienna, Austria; ^2^ Konrad Lorenz Forschungsstelle, Core Facility for Behaviour and Cognition, University of Vienna, Grünau im Almtal, Austria; ^3^ Department of Zoology, Stockholm University, Stockholm, Sweden

**Keywords:** early-social environment, brood size, attention response, common ravens

## Abstract

Social competence, i.e. defined as the ability to adjust the expression of social behaviour to the available social information, is known to be influenced by early-life conditions. Brood size might be one of the factors determining such early conditions, particularly in species with extended parental care. We here tested in ravens whether growing up in families of different sizes affects the chicks' responsiveness to social information. We experimentally manipulated the brood size of 13 captive raven families, creating either small or large families. Simulating dispersal, juveniles were separated from their parents and temporarily housed in one of two captive non-breeder groups. After five weeks of socialization, each raven was individually tested in a playback setting with food-associated calls from three social categories: sibling, familiar unrelated raven they were housed with, and unfamiliar unrelated raven from the other non-breeder aviary. We found that individuals reared in small families were more attentive than birds from large families, in particular towards the familiar unrelated peer. These results indicate that variation in family size during upbringing can affect how juvenile ravens value social information. Whether the observed attention patterns translate into behavioural preferences under daily life conditions remains to be tested in future studies.

## 1. Introduction

The social intelligence hypothesis posits that species living in complex social systems should evolve cognitive abilities to cope with the challenges derived from social life, like the formation and maintenance of differentiated social relationships and extended social networks [1–3]. Within a given species (or population), individuals are expected to vary in their ability to deal with and respond to social information, which is commonly referred to as ‘social competence'. Following Oliveira [4], social competence can be defined as an individual's ability to adjust and optimize the expression of its social behaviours according to the surrounding social information. As such, social competence can be viewed as an adaptive trait that varies between individuals because of heritable phenotypic differences, but also in response to differences in environmental conditions [5].

It is well known that epigenetic factors in early life such as rearing conditions, quantity and quality of parental care, and the presence or absence of peers—subsumed under the term social experience—can alter ontogenetic pathways and shape individual life-history trajectories [5–7]. Notably, social deprivations or stress experienced during upbringing can have detrimental consequences on the development of individual social behaviour. For instance, socially deprived and/or stressed individuals tend to engage in fewer relationships, show a lower propensity to gregariousness, increased aggressiveness, a reduced acquisition of social knowledge, and/or less elaborated strategies to access resources. Such effects have been described across taxa, i.e. in humans [8,9], non-human primates [10–12], rodents [13–15], birds [16,17] and fish [18,19]. Note that low social competence might arise from difficulties in detecting the social information (i.e. reduced social responsiveness) or from inappropriate response to this information [20].

Compared to the wealth of deprivation studies, where key social partners such as mothers or carers are lacking, relatively few studies have experimentally investigated effects of natural group size variation during upbringing, e.g. due to different number of offspring or helpers (for species engaging in cooperative breeding). A recent study on wild zebra finches [21] showed that being reared in enlarged broods results in less choosy and more central individuals in associations networks and a greater gregariousness during foraging. On the mechanistic level, these patterns were explained with increased sibling competition and thus increased stress in enlarged broods [21], as predicted by the developmental stress hypothesis [16]. Originally formulated in the context of song learning [22–24], and then applied to social learning and social behaviour in general [16,17], the developmental stress hypothesis states that competition for resources during upbringing results in increased stress which affects the development of individuals' brain structures and behavioural choices later in life.

Besides stress increment, large groups in early life might also represent enriched social environments and potentially foster the development of socio-cognitive skills, positively affecting social competence later in life. In line with this assumption, cooperative breeding cichlids that were raised in larger groups later expressed more adequate social behaviours during hierarchy formation than those raised in small groups, thereby increasing their chances to be tolerated on dominants' territories and their survival [25]. Communal rearing in mice (i.e. mothers rearing their pups in a single nest, which is a typical feature under field conditions) when compared with the standard laboratory condition (i.e. single mother with pups) was also found to affect the offspring's social responsiveness and the adequacy of expressed social behaviour to context. Specifically, male mice reared in communal nest showed a quicker establishment of hierarchy and acquisition of a dominance status [26]; increased selectivity when displaying aggressiveness [27]; and increased anxiety-like behaviour but only when being socially isolated [28]. In female mice, however, communal rearing seems to diminish responsiveness to social cues, with reduced reaction to novelty [29]. This suggests that an enriched social environment might not always potentiate social competences and mediate them differently across sexes. Overall, these findings on birds, fish and rodents strongly support the (hardly tested) assumption that the natural range of variation in early-life experience, like small or large broods in birds, can be sufficient to affect individuals' social competences, and in particular responsiveness to social information, later in life. What is yet unclear, however, is what guides the individuals' decision, i.e. do offspring from large broods have difficulties in detecting social information, or do they value social information differently from birds from small broods?

Common ravens, *Corvus corax*, are long-term monogamous birds, renowned for their elaborated socio-cognitive skills [30,31]. Pairs defend territories for breeding and show an extended period of bi-parental care [32,33]. After dispersal (three to six months after fledging), juveniles join non-breeder groups that tend to form for foraging, socializing and roosting [34,35]. Non-breeder groups show an open composition, with moderate to high degrees of fission–fusion dynamics [36]; yet, they are structured by social relationships [37] and individual preferences for particular sites [38,39]. During the non-breeder stage, ravens profit from others in finding and/or accessing food [40,41] and predator protection [42]; however, they also face high competition for resources and partners [43]. Hence, living in these groups likely depicts socio-cognitive demanding situations. This assumption has been supported by observational and experimental findings in wild and captive ravens, showing flexible behavioural responses about, for example, when to call at food [44], with whom to cooperate [45], when to intervene in conflicts and whom to join [46], when to engage in post-conflict affiliation [47,48], and when to disrupt others' bonding attempts [49]. All these manoeuvres likely rest on paying selective attention to social information, categorization of group members and/or recognition and memory of individuals and their social relationships, as demonstrated in playback experiments on subadult and adult ravens [50,51]. Considering the complexity of the non-breeder stage, we would expect ravens to develop such competences early in life. Specifically, juveniles might already start learning to recognize and to remember individuals and their social relations while growing up in their family. Family size in ravens naturally ranges from three to seven individuals (two parents and one to five chicks). This offers an ideal opportunity to test whether growing up with a varying number of siblings affects the development of individual social competence, later in life. As the competition for parental resources as well as the social information available to young ravens likely increases with sibling number, birds from small and large families should vary in their responsiveness to social information at dispersal, when they leave their family and start interacting with other peers. The adequacy of response could be expressed in whether or not they orient towards the stimuli of interest (detection) and/or in varying the duration of attention according to the social category of stimuli (value).

We here experimentally tested the effect of brood size variation on juvenile ravens' ability to discriminate between calls from same-aged peers of different social categories in the early non-breeder state. Simulating the natural breeding dynamics and brood size variation of ravens, we manipulated brood size of captive breeding pairs over three consecutive years, creating ten small families (seven with two chicks and three with one chick) and six large families (four with three chicks and two with four chicks) in a cross-design. After fledging in early May, juvenile ravens stayed in their families until mid-July. They were transferred into one of the two non-breeder aviaries, where they formed two similar-sized groups of same aged peers. After five weeks of socialization in those non-breeder groups, we tested birds individually in a playback design with calls from a sibling (with whom they grew up and were transferred with into the non-breeder group), a familiar non-sibling (unrelated individual with which they were housed in a captive non-breeder group) and an unknown non-sibling (unrelated individual housed in the other non-breeder group, never encountered). Following Brandl *et al.* [21], we hypothesized that ravens brought up in large families experienced more sibling competition and thus increased developmental stress as compared to ravens brought up in small families. We thus expected ravens from large families to be less choosy/more open in with whom to interact, which should result in similar responses to the different played back calls, irrespective of the social category. We also reasoned that ravens brought up in large families learn to divide their attention between more partners, whereas ravens brought up in small families had their attention focused on a single sibling. Accordingly, we expected ravens from small families to show more selective responses to calls from individuals they know, notably their sibling and potentially also a familiar peer they have recently been housed with. An alternative hypothesis would be that experience with different siblings could give birds from large families an advantage in detecting social information; in this case, we would predict birds from large families to be more skilled in differentiating between callers of different social categories than birds from small families. Taken together, we expected the offspring from large and small broods to detect/value social information differently.

## 2. Material and methods

### 2.1. Study subjects and housing

We worked with 28 juvenile ravens from 13 captive families throughout three consecutive breeding seasons (2018–2020). Families were generated from nine captive breeding pairs (electronic supplementary material, table S1). All families were housed separately from each other at Haidlhof Research Station (four pairs), Konrad Lorenz Research Center (four pairs) and Zoo Vienna (one pair) in spacious aviaries (80–120 m^2^). See electronic supplementary material, table S1, for more details on families' composition and location. Families were housed in separated aviaries and although those families located in the same location could hear each other, offspring from different families never met or interacted physically in the family phase. Throughout the consecutive breeding seasons, we manipulated the brood size by removing and/or replacing eggs to create same sized clutches of four eggs, from which either all four (large family) or only two eggs were fertile (small family). Which pair received a large or small brood treatment was randomly allocated in the first year and then changed across years following a cross-design (e.g. pairs with a large brood in year one were treated as a small brood in year two). In some cases, the actual offspring number per pair deviated from our egg manipulation. Typically because an egg did not hatch or a chick died in the first days, resulting in an uneven number of siblings (one juvenile: small family; three juveniles: large family). All juveniles were marked with coloured rings for individual identification before fledging.

Juvenile ravens were raised from hatching (late March–early April) to approximately 10 weeks post fledging (early May) by their parents (family phase). In mid-July of each year, all chicks were taken out from their parents' aviary on the same day and transferred to one of the two non-breeder aviaries of the Konrad Lorenz Research Center, in the area of the Cumberland Wildpark Grünau. Captive non-breeder groups ranged in size between six and nine individuals. To compose each group, we control for sibling number, transferring a maximum of two siblings from the same family per group. As a result: single juveniles from one-juvenile families (small) were transferred alone in one group; siblings from two-juveniles families (small) were transferred together in the same group; siblings from three-juveniles families (large) were split in one and two chicks in each group; while siblings from four-juveniles families (large) were split in two dyads of two in each group. Note that we excluded single juveniles with no sibling in their non-breeder group from the study, resulting in a total of 28 subjects (13 from small families; 8 females and 5 males and 15 from large families; 6 females and 9 males; electronic supplementary material, table S1). The two non-breeder aviaries were of similar size (60 m^2^) and equipped similarly with natural ground cover (gravel, sand, grass), wooden perches, shallow pools for bathing and roofs for sun and rain protection. They were located 1.5 km apart, separated by dense forest areas, preventing birds of the two peer groups from being in any visual or acoustic contact. Juveniles in both groups were fed twice a day with a mixture of pellets, meat, vegetables and fruits and they had ad libitum access to water. The ravens stayed in the non-breeder groups for a total of six weeks, before being released in the wild by the beginning of September. In the week before release, juveniles were individually taken out from their group for being measured, blood sample and marked with rings from the Austrian Ornithological Center and coloured wing tag, and equipped with a GPS logger. We scheduled our playback experiment in the same week to make use of the birds' separation from the group for this marking procedure.

### 2.2. Experimental setting

After being measured and marked, juveniles were transferred to an experimental aviary (2 m^3^), which was temporally set up in a remote woodland part of the park, more than 2 km away from any keeping aviaries. In the experimental aviary, the test subject was provided with food and water ab libitum and it remained undisturbed by humans for about 20 h, including overnight, before playback experiments were conducted. Then the playback was conducted. The experimental aviary was equipped with two perches positioned at the same height (1.5 m above ground), both were perpendicular to a back wall which was covered by an opaque plastic sheet. Broadcasting stimuli came from a loudspeaker located behind the opaque back wall at a distance of 2 m from the aviary. On the opposite side, a GoPro Hero 7 was mounted on a tripod for video recording of the subject's behaviour.

### 2.3. Acoustic recordings and playback experiments

We used food-associated calls known for their individual signature [41,52]. Calls were recorded in the last week of the family phase prior juveniles' transfer to the non-breeder groups. Standing outside the aviary, we individually identified juveniles by their coloured rings and recorded their calls using a shot-gun microphone (Sennheiser ME-67) plugged into a Tascam DR-100mkII voice recorder (.wav format, 44.1 kHz, 16bit-rate).

During the playback experiment, each subject was exposed to two consecutive calls (separated by 0.3 s) from each of the three social categories: sibling (related individual with whom the subject was brought up and then housed in the non-breeder group), familiar individual (a random unrelated individual with whom the subject was housed in the non-breeder group) and unknown individual (a random unrelated individual from the other non-breeder group, never encountered by the subject). Note that for unknown individuals, calls were collected from a different family coming either from a different or same research station. However, since families were housed in single aviaries, these juveniles had never met and thus interacted. The broadcasting order for caller categories was randomized across tested subjects and a 6 min silence pause occurred between the exposure to each category of call. Two of the 28 subjects received two unknown and one familiar unrelated calls (instead of one call of each type) due to missing recordings from their sibling. All calls' amplitude was standardized using Audacity software (https://www.audacityteam.org/) to match an identical broadcasting volume of 67 dB measured at 2 m distance (Sound Level Meter RadioShack, model 3300099, A-weighting, fast response). Calls were played back in .wav format using a digital music player (Musrun k188) connected to a loudspeaker (JBL xtreme, frequency response 70–20 000 Hz) and the loudspeaker was placed in a blind side of the experimental aviary, this being the opposite site to where the camera recorded subjects' behaviours.

### 2.4. Behaviour responses and video coding

From the video recordings, we coded the subjects' behaviour in the minute before the stimulus was played (baseline phase) and in the minute right after the stimulus was played (test phase). Videos were coded using the free software Solomon Coder (https://solomon.andraspeter.com/). Specifically, we scored the number and duration of orientation responses towards the opaque wall (‘looks’: lateral head position, facing with their beak in the direction of the loudspeaker). It results in two behaviour measurements: ‘Duration of head turns' (seconds) and ‘Number of looks'. The majority of video sequences were coded by a single coder (I.M., 82%), who was blind to the hypothesis and the caller's identities. About 20% were coded by M.G.-A. (inter-observer reliability between the two coders: Cohen's kappa, *K* = 0.904, *p* < 0.001; R package ‘irr' [53]). We used delta values as response variable in our statistical analyses by subtracting the measurements (frequencies and durations) of the 1 min baseline phase from those of the 1 min test phase.

### 2.5. Statistical analysis

We investigated how brood size affected ravens' attentiveness to callers from different social categories. To do so, we ran linear and generalized linear mixed effect models, respectively using the (i) ‘Duration of head turns' and (ii) ‘Number of looks' as response variables using the functions *lmer* and *glmer* (Poisson error distribution, *log link* function) within R package ‘lme4’, respectively [54]. In both models, we included ‘Brood size’ (categorical: small, large), ‘Caller class' (categorical: sibling, familiar, unknown), ‘Sex' of the subject (categorical: male, female), and the two interactions between ‘Brood size: Caller class' and ‘Sex: Caller class' as fixed factors. ‘Subject' nested within ‘Family identity' were included as random factors. To test for the overall significance of each interaction, we ran full–reduced model comparisons, between the above-mentioned full model and reduced models (lacking each interaction). We also ran a full–null model comparison between the full model and the null model lacking all fixed factors (but including the random factors). For model comparisons we used the *anova* R test function and reported AIC, degree of freedom, χ^2^ and *p*-values to assess significance of the interaction. For ‘Caller class', we ran *post hoc* comparisons applying Tukey's contrasts (*glht* function, ‘multcomp' R package [55]). We also ran *post hoc* comparisons for the interaction terms ‘Brood size: Caller class' and ‘Sex: Caller class' using the functions ‘emmeans; pairwise comparison’ and ‘contrast’ within the ‘emmeans' R package [56] to assess estimated marginal means and report associated *p*-values. For the linear model, the normality of the residuals was confirmed using the Shapiro–Wilk normality test (function *shapiro.test*, ‘stats' R package). All our statistical analyses were conducted in R software, v. 4.1.1. [57], with a significance threshold set at *α* = 0.05.

## 3. Results

**Figure 1.**
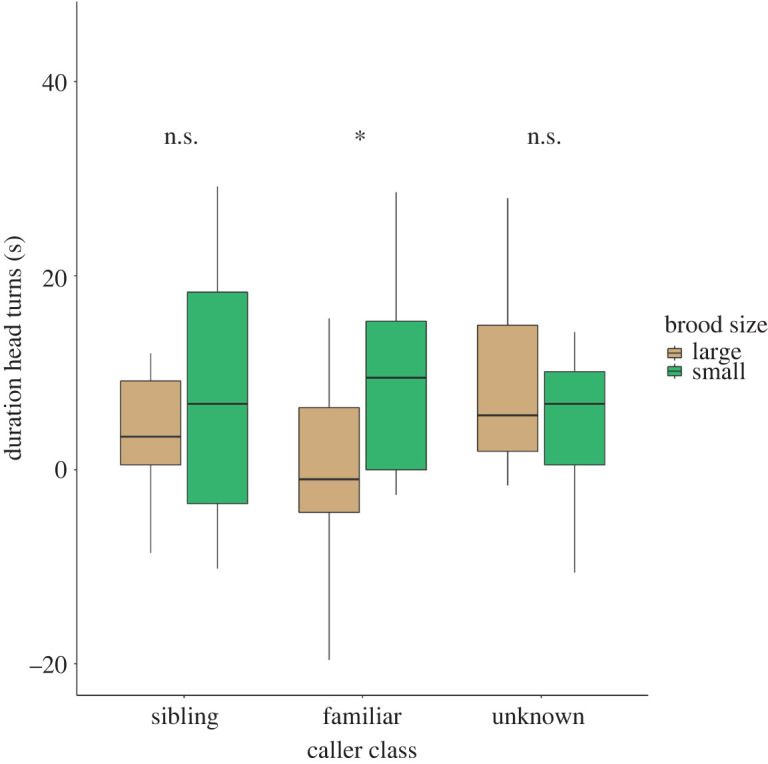
Attention responses of tested subjects towards the three different caller categories. The figure shows the Δ values (response − baseline) of ‘Duration of head turns' in seconds, towards the three social categories and coloured by ‘Brood size'. The resulting significance from generalized linear mixed effect model and *post hoc* analysis is indicated as * when *p* < 0.05.

The full–reduced and full–null model comparisons for the Duration of head turns supported the inclusion of the interactions (Caller class: Brood size and Caller class: Sex) in the full model, table 1*b*. As a result, the individual rearing background (Brood size) had an effect on juveniles' attention responses; however, this effect was mediated by the caller class, with a greater difference between large and small families for calls of familiar individuals (figure 1 and table 1). Specifically, emmeans contrasts based on the full model revealed that small-family juveniles looked significantly longer in the direction of the loudspeaker than large-family juveniles when broadcasting familiar calls; while this difference was not significant for other caller classes (see contrasts in table 1*d*). Interestingly, no significant differences were found when comparing ‘duration of head turns' between caller class categories within each rearing group (small and large brood size, table 1*e*). Although non-significant, the sex appeared to a lesser extent to influence responsiveness for certain caller class (see overall significance of the interaction term and *post hoc* comparisons in electronic supplementary material, table S3). Descriptively, females seemed to look longer in the direction of the loudspeaker than males when broadcasting familiar calls (see electronic supplementary material, figure S1), irrespectively of the caller sex (see electronic supplementary material, figure S5). We found no significant effect of the tested predictors (‘Brood size’, ‘Caller class’, ‘Sex’, ‘Brood size: Caller class' and ‘Sex: Caller class’) on the ‘Number of looks’ (see electronic supplementary material, table S2 and figures S2 and S3).

**Table 1.** (*a*) Summary of the linear mixed model results containing the interactions ‘Brood size: Caller class' and ‘Sex: Caller class' to explain the attentive response (duration of head turns) of juvenile ravens. (*b*) Full–reduced and full–null model comparisons using the ANOVA test, reporting the degree of freedom, AIC, χ^2^ and *p*-values. (*c*) *Post hoc* comparison using Tukey contrast comparison for ‘Caller class'. (*d*) *Post hoc* comparisons of estimated means between ‘Brood size' for each ‘Caller class', using emmeans package. (*e*) *Post hoc* comparisons of estimated means between ‘Caller class' for each ‘Brood size', using emmeans package. Values of *p* in bold indicate significance lower than 0.05.

full model	estimate ± s.e.	CI	*t*-value	*p*-value
(*a*)				
*intercept*	6.18 ± 4.07	−1.93–14.29	1.52	0.133
caller class (*sibling*)	−2.89 ± 5.51	−13.87–8.08	−0.53	0.601
caller class (*unknown*)	0.01 ± 5.42	−10.78–10.81	0	0.998
brood size (*small*)	11.21 ± 4.51	2.23–20.19	2.49	**0**.**015**
sex (male)	−8.21 ± 4.51	−17.19–0.77	−1.82	0.073
caller class (*sibling*) × brood size (*small*)	−5.73 ± 6.2	−18.09–6.63	−0.92	0.359
caller class (*unknown*) × brood size (*small*)	−16.34 ± 5.94	−28.17 to −4.50	−2.75	**0**.**008**
caller class (*sibling*) × sex (*male*)	4.69 ± 6.17	−7.61–16.99	0.76	0.449
caller class (*unknown*) × sex (*male*)	13.89 ± 5.94	2.04–25.74	2.34	**0**.**022**
**full to reduced and null models comparison, ANOVA test**				
(*b*)				
*full model* caller class × brood size + caller class × sex	AIC = 675.90			
*reduced model* caller class + brood size + caller class × sex	AIC = 679.32, *χ*^2^ = 7.43, d.f. = 2, *p*-value = 0.02439			
*reduced model* caller class × brood size + caller class + sex	AIC = 677.15, *χ*^2^ = 5.25, d.f. = 2, *p*-value = 0.072			
*null model*	AIC = 676.81, *χ*^2^ = 16.91, d.f. = 8, *p*-value = 0.03105			
**multiple comparisons of means: Tukey contrasts**				

	**estimate ± s.e.**	**z*-*value**	***p*-value**	
(*c*)				
sibling–familiar non-sibling	−2.895 ± 5.50	−0.526	0.859	
unknown–familiar non-sibling	0.01 ± 5.41	0.002	1	
unknown–sibling	2.91 ± 5.47	0.531	0.856	

**emmeans contrasts**	**estimate ± s.e.**	**d.f.**	***t*-ratio**	***p*-value**
(*d*)				
**caller class = familiar non-sibling**				
brood size (large–small)	−11.21 ± 4.93	84.5	−2.276	**0**.**025**
**caller class = sibling**				
brood size (large–small)	−5.48 ± 5.15	85.3	−1.065	0.29
**caller class = unknown**				
brood size (large–small)	5.13 ± 4.83	81	1.062	0.29

**emmeans contrasts**	**estimate ± s.e.**	**d.f.**	***t*-ratio**	***p*-value**
(*e*)				
**brood size = large**				
familiar non-sibling–sibling	0.549 ± 4.36	62.5	0.126	0.9913
familiar non-sibling–unknown	−6.958 ± 4.36	62.5	−1.598	0.2542
sibling–unknown	−7.507 ± 4.36	62.6	−1.723	0.2049
**brood size = small**				
familiar non-sibling–sibling	6.277 ± 4.89	65.2	1.283	0.4098
familiar non-sibling–unknown	9.377 ± 4.51	64.2	2.081	0.1019
sibling–unknown	3.100 ± 4.85	71.0	0.640	0.7988

## 4. Discussion

In the present study, we tested the responsiveness of juvenile ravens from small or large families to social information provided in food-associated calls. We experimentally show that, irrespective of their rearing history, juvenile ravens respond to playbacks of food calls of same-aged peers at the time of dispersal (about five months of age). We also show that family size has an effect on the birds' responsiveness, but only for a certain type of social category, i.e. for familiar individuals they have been housed with, in the previous five weeks (non-breeder stage). Indeed, while family size did not seem to affect juveniles' responsiveness to calls from a sibling (they have also been housed with) or an unknown individual (they have never encountered before), juveniles reared in small families paid longer attention to acoustic cues from familiar individuals than juveniles reared in large families. These results hint in the direction that differences in upbringing affect the social competence of ravens at dispersal, as birds from small and large families seem to value social information differently.

Our findings are partly in line with the developmental stress hypothesis [16,17], predicting that individuals from large families should be less choosy in with whom to interact. In line with this prediction, ravens from large families were equally attentive to the played back calls, irrespective of the callers' social category. However, ravens from small families showed the same pattern; contrary to our prediction, they were neither selective among the caller categories nor more focused on their sibling (table 1*e*). Possibly, ravens at that early age cannot yet fully pick up on the social information encoded in food calls, making it difficult for them to discriminate between played back callers. That the tested birds have not yet developed their full cognitive capacity is supported by behavioural studies on the ontogeny of food caching [58] and gaze following [59], indicating a cognitive step in the ravens' development at the end of their first summer (September/October), which might go together with becoming more selective to social cues [60]. Our study was scheduled to match the situation of wild ravens at our field site (family phase until early summer, local dispersal and meeting of same-aged peers in summer, integration into non-breeder population in late summer/early autumn). Accordingly, the playbacks were carried out in late August, which might have been too early. We do not know of any other study testing for social discrimination in juvenile ravens, or other corvids at a comparable age, and further studies during this sensitive period would be required to better understand possible developmental effects.

Aside the cognitive development, it is well known that young ravens are generally attracted to raven calls [61,62]. Particularly food calls from same-aged conspecifics might be highly salient to them, as those indicate the opportunity to join others at food [61] and, potentially, to socialize with them after feeding [63]. A genuine interest in same-aged peers could thus explain the similar levels of attention shown to callers of different social categories in our experiment. This salience argument would also help explaining our main finding on the effect of upbringing, i.e. that birds from small families generally attended more to playbacks of food-associated calls of familiar individuals than birds from large families. Possibly, ravens coming from small families were more interested in meeting same-aged peers as compared to ravens from large families. Indeed, ravens start to form affiliative relationships already in their first autumn [60], when they face the challenge of integrating into non-breeder groups [64]. Siblings are preferred partners at that stage, providing social support during and post-conflict [48]. It is conceivable that birds from small families should be more interested in enlarging their social network including known peers than birds from large families, who can already rely on support from several siblings.

It must be noted that even though we do find different attention responses of ravens raised in small and large families, and hence an effect of our brood size manipulation (table 1*d*), we do not yet know what specifically has caused this effect in early life, differences in the parents' behaviour (e.g. feeding rates) and/or differences in the offspring's behaviour (e.g. aggression rates). Interestingly, ravens from small and large families differed strongest in the response to calls from familiar non-siblings, indicating that unrelated but familiar individuals were more salient to ravens from small families compared to ravens from large families. We found no noticeable difference between small versus large families for other types of calls, and this result held even when differentiating unknown callers coming from different versus from the same research site who might have had some acoustical (but no visual and physical) contact during upbringing (see electronic supplementary material, figure S4).

Coming back to the developmental stress hypothesis, similarly as described for song learning [24,65], it is plausible that more acute stress experienced during upbringing in large families could have affected the development of certain brain areas of large-family juveniles and potentially resulted in lower preferences for attending to social information later as juveniles. Although we have not analysed stress level of our study subjects, we recently showed that parental care investment varies with brood size, whereby chicks in large families receive significantly less care (feeding and affiliations) than chicks raised in small families, which might increase stress [66]. In this respect, our results could align with those on zebra finches, which showed different social learning strategies depending on the levels of developmental stress induced during upbringing [16,17]. Additionally, large-family juveniles could have experienced a stress-related phenomenon due to separation from other siblings, since no more than two siblings were transferred into the same non-breeder groups. While we do know that separation and reunion events can induce stress-related behaviours and hormonal responses, these typically last no longer than a few days [67]. Future studies need to investigate in more detail the mechanisms underlying the long-lasting effects of varying offspring number at upbringing and the first peer group formation on the development of ravens' social competences.

Finally, although non-significant, our analyses also suggest that upbringing conditions might affect ravens of both sexes differently, which would fit with the findings in domestic mice [26,27,29]. Indeed, while the effect of upbringing was particularly salient for familiar calls, we also see descriptively that females tended to be more attentive to these calls than males, whereby the sex of the caller had no effect on the response of either males or females (see electronic supplementary material, figure S5). Note that males and females were equally represented in the dataset for both family sizes. Future studies should thus aim to consider the possibility of differential early social environment effects in the two sexes.

Taken together, our findings support the assumption that offspring from small families come to value social information differently from birds from large families. By contrast, having experience with different number of siblings did not result in any apparent advantage for large-family juveniles over small-family juveniles in detecting information encoded in calls, at least not in our playback experiment. This could be different in alternative test settings, when birds are required to interact with one another, for instance in a separation–reunion setting (see [67]). Future studies should attempt testing both the response to acoustic cues and the willingness to interact with the caller. Furthermore, our findings need to be corroborated in older birds, also tested under ecologically relevant conditions [21]. Indeed, we plan to track our study subjects under free-flight conditions until adulthood (i.e. four years), which should allow us to investigate how the environment experienced during upbringing shapes the ravens' social behaviour, association patterns, and network positions at different stages or their life as non-breeders and, eventually, their social competence as adults.

## Data Availability

Dataset and R script files are available within Dryad: https://doi.org/10.5061/dryad.3bk3j9kmj [68]. The data are provided in electronic supplementary material [69].
